# Comparison of the use of a clinically implemented deep learning segmentation model with the simulated study setting for breast cancer patients receiving radiotherapy

**DOI:** 10.2340/1651-226X.2024.34986

**Published:** 2024-06-20

**Authors:** Nienke Bakx, Maurice van der Sangen, Jacqueline Theuws, Johanna Bluemink, Coen Hurkmans

**Affiliations:** a Department of Radiation Oncology, Catharina Hospital, Eindhoven, The Netherlands; b Departments of Applied Physics and Electrical Engineering, Technical University Eindhoven, Eindhoven, The Netherlands

**Keywords:** deep learning, breast cancer, auto-segmentation, clinical

## Abstract

**Background:**

Deep learning (DL) models for auto-segmentation in radiotherapy have been extensively studied in retrospective and pilot settings. However, these studies might not reflect the clinical setting. This study compares the use of a clinically implemented in-house trained DL segmentation model for breast cancer to a previously performed pilot study to assess possible differences in performance or acceptability.

**Material and methods:**

Sixty patients with whole breast radiotherapy, with or without an indication for locoregional radiotherapy were included. Structures were qualitatively scored by radiotherapy technologists and radiation oncologists. Quantitative evaluation was performed using dice-similarity coefficient (DSC), 95th percentile of Hausdorff Distance (95%HD) and surface DSC (sDSC), and time needed for generating, checking, and correcting structures was measured.

**Results:**

Ninety-three percent of all contours in clinic were scored as clinically acceptable or usable as a starting point, comparable to 92% achieved in the pilot study. Compared to the pilot study, no significant changes in time reduction were achieved for organs at risks (OARs). For target volumes, significantly more time was needed compared to the pilot study for patients including lymph node levels 1–4, although time reduction was still 33% compared to manual segmentation. Almost all contours have better DSC and 95%HD than inter-observer variations. Only CTVn4 scored worse for both metrics, and the thyroid had a higher 95%HD value.

**Interpretation:**

The use of the DL model in clinical practice is comparable to the pilot study, showing high acceptability rates and time reduction.

## Background

The treatment planning workflow for breast cancer radiotherapy contains multiple steps which are mostly performed manually, including segmentation of target volumes and surrounding organs. This manual nature introduces intra- and interobserver variability (IOV), due to for example varying experience and guideline interpretation of treatment planners and radiation oncologists (ROs) [1, 2]. Besides, it is a time-consuming part of the workflow, and therefore multiple methods to automate this step were introduced in recent years.

First, atlas-based auto-segmentation was introduced [3, 4]. More recently, deep learning (DL) models showed the most promising results regarding segmentation quality and time-saving [5–10]. However, most studies only report results of these models in a retrospective or pilot setting. A study on clinical implementation of DL planning for radiotherapy already showed that this might not sufficiently reflect the real-world setting [11]. Therefore, one should monitor the performance of the DL model in clinical use, regarding its usage, results, adjustments, and other relevant factors [12].

Previously, in our clinic, a pilot study was performed for an in-house trained DL segmentation model for locoregional breast cancer radiotherapy, after which the model was implemented in the clinical workflow [13]. This study aims to assess if differences in performance or acceptability arise after clinical implementation, by evaluating the use of the model in the real-world clinical setting and comparing it to the previous results of the pilot study. By performing this study, an extensive validation of the DL model and its implementation is performed. This work therefore meets the demand for validation of such models as reported by several studies to overcome the barriers to clinical implementation [12, 14, 15].

## Materials and methods

### Data

The in-house trained DL segmentation model is used in our clinic, Catharina Hospital Eindhoven, since November 2022 for both left- and right-sided breast cancer. The DL model automatically generates contours for 11 regions of interest (ROIs), including the clinical target volumes (CTVs) (breast [CTVp], axillary lymph node levels 1–3 [CTVn1, CTVn2, and CTVn3] and supraclavicular lymph node [CTVn4]) as well as all involved organs at risk (OARs) (heart, left/right lung, esophagus, thyroid, and humeral head). Model training was performed in cooperation with RaySearch (RaySearch laboratories, AB, Sweden), using a 3D U-net architecture [16]. More information on the training procedure can be found in Ref. [13].

The DL model is used for patients treated with either partial or whole breast radiotherapy, with or without an indication for inclusion of either lymph node levels 1 and 2, or levels 1 to 4. The inclusion of these different patient groups means that either all 11 ROIs or a subset of them are used for these patients.

The evaluated clinical workflow consists of several steps, performed in RayStation 10B-SP1. First, the radiotherapy technologist (RTT) loads a protocol-specific structure template, after which the involved ROIs are automatically generated. The RTT checks the OARs and corrects them when needed. Then, the RO checks and corrects the CTVs and performs an extra check on the OARs. Finally, the planning target volumes are automatically derived from the CTVs. In a daily meeting, all new patient segmentations and treatment plans are discussed with multiple ROs and a medical physicist. This process is equal to the process of manual segmentation, where empty structures are loaded, which are then segmented by the corresponding RTT or RO.

In this study, 15 RTTs and 7 ROs with varying experience participated, in contrast to 5 RTTs and 5 ROs in the pilot study. Sixty patients were included, treated between November 2022 and March 2023. Contouring of either OARs or CTVs, or both, was timed and structures were scored as described further in the text. Contours which were not scored or timed were still included in the quantitative analysis. The total number of patients included for all ROIs is listed in Table 1.

**Table 1:** The number of patients included in the evaluation for each region of interest.

ROI	Number of cases segmented	Number of cases timed
CTVp	46	26
CTVn1	30	15
CTVn2	32	17
CTVn3	19	10
CTVn4	19	10
Heart	60	54
Left/right Lung	60	54
Esophagus	29	24
Thyroid	20	18
Humeral head	32	29

ROI: region of interest.

### Evaluation

The clinical use of the DL model was evaluated qualitatively and quantitatively. The RTTs and the ROs were asked to time the process in which they load, check, and correct the contours. In addition, they scored the contours using a 3-point Likert scale: (1) clinically acceptable, no correction is needed; (2) not clinically acceptable, but can be used as a starting point while still saving time; (3) not clinically acceptable, and cannot be used as a starting point. Statistical significance of the time and scoring between the pilot study and clinical phase was assessed using the Wilcoxon Rank Sum Test and Mann–Whitney *U* Rank Test, respectively.

Besides, three quantitative metrics were used to compare the DL generated contours with the corrected contours. These metrics are the Dice Similarity Coefficient (DSC), 95^th^ percentile of the Hausdorff Distance (95%HD), and the surface DSC (sDSC) [17]. The first two quantitative metrics were also compared to IOV from two other studies [9, 10].

## Results

Time needed for checking and correcting ROIs is listed in Table 2. Since the pilot study only included patients with an indication for locoregional radiotherapy, no reference time is available for the segmentation of only CTVp. For both target volume subgroups, more time was needed to check and correct when compared to the pilot study, although time reduction is still statistically significant, with 41% and 33% for the CTVn1–2 and CTVn1–4 subgroups respectively, when compared to manual segmentation. Furthermore, it was only found to be statistically significantly slower compared to the pilot study for the CTVn1–4 subgroup. For the OARs, the time reduction increased from 58% and 39% to 64% and 47%, for the subgroup without and with thyroid, respectively.

**Table 2:** Time needed (mean ± standard deviation, mm:ss) by RTTs and ROs to check and correct (a subset of) ROIs using the DL model (column 1). Column 2 and column 4 display results of a previously performed pilot for comparison [13]. P-values indicate statistical significance between clinical phase and pilot study.

ROIs	DL segmentation (clinical phase)	Manual segmentation (pilot study)	*p*	DL segmentation (pilot study)	*p*
CTVp	09:43 ± 05:42	-	-	-	-
CTVp and CTVn1–2	21:00 ± 06:29	35:53 ± 13:54	0.03	13:50 ± 05:42	0.08
CTVp and CTVn1–4	30:32 ± 13:14	45:49 ± 10:59	0.04	17:15 ± 04:47	0.02
Heart, lungs, esophagus, humeral head	06:51 ± 02:26	18:57 ± 06:04	<0.01	07:54 ± 01:56	0.22
Heart, lungs, esophagus, humeral head, thyroid	08:13 ± 03:27	15:33 ± 03:55	<0.01	09:31 ± 02:12	0.16

DL: Deep learning.

**Figure 1:**
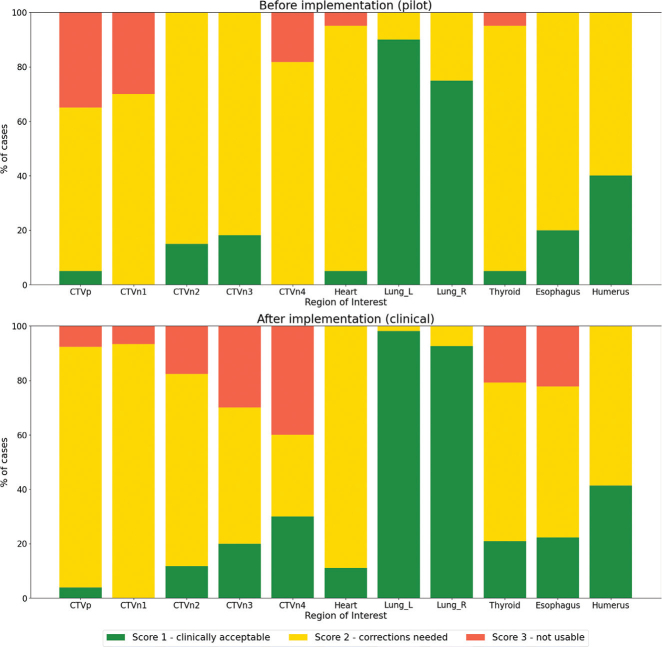
Qualitative scoring by the RTTs and ROs for all regions of interests, before and after implementation of the deep learning model.

Figure 1 shows the distribution of the qualitative scores assigned by the RTTs and ROs. In total, 93% of all contours were scored as clinically acceptable or usable as a starting point after implementation, comparable to 92% found in the pilot study. In almost all cases after implementation, the segmentation of the OARs received a score 1 or 2, except for nine cases for the thyroid and esophagus. In one case, it was found that the thyroid was not automatically segmented by the DL model due to a metal artifact, requiring complete manual segmentation. Eighty-three per cent of the CTVs were scored as clinically acceptable or corrections needed, which is again comparable to 82% in the pilot study. While CTVn3 and CTVn4 were scored as not usable in respectively 30% and 40% of the cases, they were also found to be clinically acceptable without corrections in respectively 20% and 30% cases. When comparing the scores of the CTVs, a tendency towards lower scores can be observed for node levels 2 to 4, and towards higher scores for CTVp and CTVn1, although no statistically significant differences were found.

**Figure 2:**
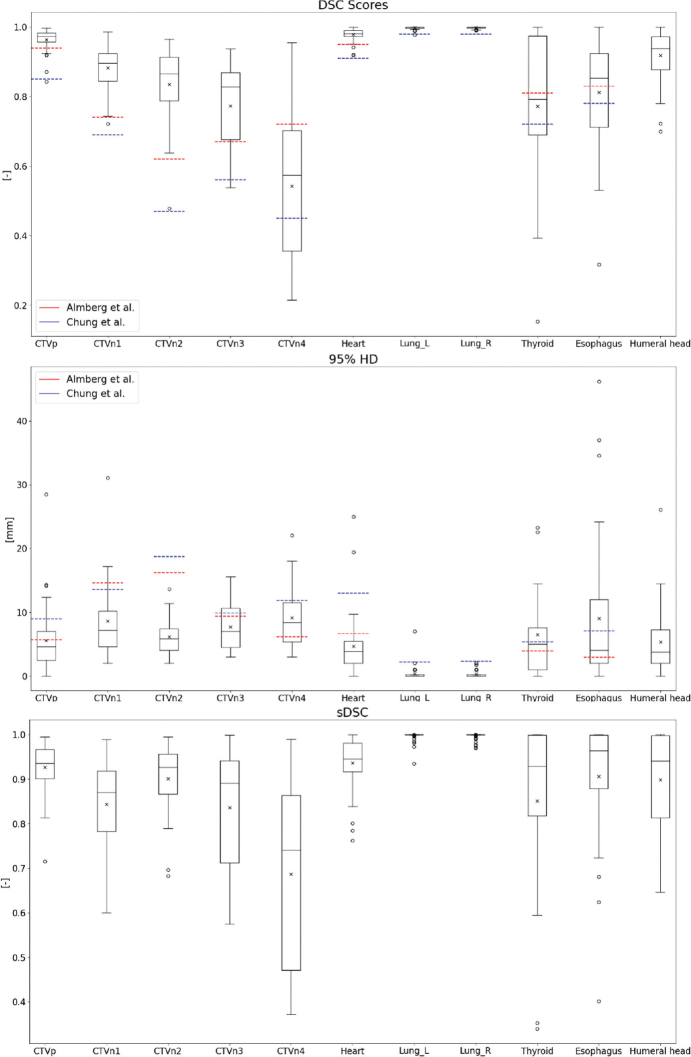
Boxplots of dice-similarity coefficient (DSC) scores (top), 95%HD (middle), and sDSC (bottom) scores of the comparison between deep learning contours and corrected contours. Horizontal lines in boxes are median values, crosses are mean values, dots are outliers. Interobserver variability values of Almberg et al. (red) and Chung et al. (blue) are indicated by horizontal lines.

Quantitative outcomes are shown as boxplots in Figure 2. Almost all ROIs have a higher mean and median DSC score and a lower 95%HD value than IOV values, indicating good performance. CTVn4 scored worse when compared to Almberg et al. for both metrics, and both thyroid and esophagus have a higher mean 95%HD than both IOV values. For the esophagus, a mean 95%HD of 2.35 ± 2.05 mm was found when only considering the overlapping part of the DL and corrected esophagus, and thus excluding differences in length.

## Discussion

This study evaluated the clinical use of a DL segmentation model for locoregional breast cancer radiotherapy, compared to a previously performed pilot study. A total of 93% of all contours in clinic were scored as clinically acceptable or usable as a starting point, comparable to 92% in the pilot study. Moreover, time saving was shown for both target volumes and OARs, when compared to manual segmentation times in the previously performed studies. Quantitative evaluation showed that almost all corrections made were within the range of the IOV found in similar studies.

Time saving is one of the endpoints of using the DL segmentation model in clinical practice, and therefore it is important to monitor. In clinical practice, more time was needed to check and correct target volumes when compared to the pilot study. This was found to be for some part caused by the fact that in clinical practice, the RO might use additional patient information, such as extra imaging or the surgical and pathological report, to determine the exact locations of the involved lymph nodes. However, in the pilot studies, such patient-specific information was not provided and thus no time was spent on this. Moreover, more ROs with different experience were included in this study, which could also explain the difference in time found. This difference in time stresses the importance of monitoring and evaluating a DL model in clinical practice.

In contrast to the target volumes, time reduction was slightly higher for the OARs in clinical practice, when compared to the pilot study, although it was not statistically significant. This result suggests that the RTTs perform less corrections on the OARs, indicating an increased trust in the outcome of the DL model. However, this result is not necessarily reflected in the qualitative scores, as the percentage of structures assigned score 1 or 2 is similar between the pilot study and clinical phase, emphasizing the subjectivity of these qualitative scores.

The number of RTTs and ROs using the DL model increased from 5 for both in the pilot phase, to respectively 14 and 7 in clinical use. The comparable results between these phases therefore indicate a good adoption of the DL model in the clinical workflow. These results are in contrast to the results found by McIntosh et al., finding a distinctive difference in perception towards the use of AI in clinical practice compared to the study setting [11]. This difference in results might be because several RTTs and ROs were involved from the start of the project, to increase support from the eventual end-users for its development and implementation.

For the quantitative evaluation, the original DL contour was compared to the corrected contour and resulting metrics were compared to IOV values of two similar studies. It could be stated that small corrections, resulting in a high DSC score or low 95%HD score, could be clinically irrelevant when these values are better than the reported IOV values, which is the case for most structures in this study. However, clinical relevance cannot be fully captured in these metrics, since a small deviation in contouring could have a significant clinical impact. Besides, the IOV values were taken from other studies, and therefore might not reflect the actual variability within our institute. To get more insight in the clinical relevance of these corrections, the dosimetric impact on the treatment plan needs to be studied, which is the next step within our implementation team.

The difference in the 95%HD for the full esophagus and cropped esophagus indicates that most corrections are made to adjust the length. However, it was found that the esophagus was not systematically made shorter or longer. Furthermore, corrections were both caudal and cranial. These results, supported by visual inspection, indicate a low consensus among the RTTs on segmentation of the esophagus.

A review session with RTTs and ROs was organized and was shown to be helpful to improve institutional interpretation and consensus on segmentation guidelines. 3D meshes of the corrected contour with a color map indicating the 95%HD to the non-corrected contour were visualized to get a better understanding of the relevance of corrections. Examples of the 3D meshes used can be found in the Supplementary material. A similar approach was recently described by Mikalsen et al., who held a plenary session to ensure internal consensus on the guidelines before evaluating a DL segmentation model [18]. Moreover, a multi-institutional comparison of manual segmentations versus DL segmentations was performed (unpublished data). This comparison showed that the ESTRO guidelines for CTVn4 were interpreted differently in our institute than others, leading to a better inter-institutional awareness of perceived differences. Thus, incorporating data from other institutes is useful to reduce inter-observer variability and increase quality of the segmentations.

In conclusion, a DL model was successfully implemented in clinical practice. Although corrections are still made, time reduction is achieved when compared to manual segmentation. Moreover, the results are similar to the pilot study, suggesting good adoption.

## Data Availability

Due to the nature of the research and due to legal supporting data is not available.

## References

[1] Li XA, Tai A, Arthur DW, Buchholz TA, Macdonald S, Marks LB, et al. Variability of target and normal structure delineation for breast cancer radiotherapy: an RTOG multi-institutional and multiobserver study. Int J Radiat Oncol Biol Phys. 2009;73:944–51. 10.1016/j.ijrobp.2008.10.034 19215827 PMC2911777

[2] Ciardo D, Argenone A, Boboc GI, Cucciarelli F, De Rose F, De Santis MC, et al. Variability in axillary lymph node delineation for breast cancer radiotherapy in presence of guidelines on a multi-institutional platform. Acta Oncol (Madr). 2017;56:1081–8. 10.1080/0284186X.2017.1325004 28534430

[3] Eldesoky AR, Yates ES, Nyeng TB, Thomsen MS, Nielsen HM, Poortmans P, et al. Internal and external validation of an ESTRO delineation guideline – dependent automated segmentation tool for loco-regional radiation therapy of early breast cancer. Radiother Oncol. 2016;121:424–30. 10.1016/j.radonc.2016.09.005 27697296

[4] Ciardo D, Gerardi MA, Vigorito S, Morra A, Dell’acqua V, Diaz FJ, et al. Atlas-based segmentation in breast cancer radiotherapy: evaluation of specific and generic-purpose atlases. Breast. 2017;32:44–52. 10.1016/j.breast.2016.12.010 28033509

[5] Men K, Zhang T, Chen X, Chen B, Tang Y, Wang S, et al. Fully automatic and robust segmentation of the clinical target volume for radiotherapy of breast cancer using big data and deep learning. Phys Med. 2018;50:13–9. 10.1016/j.ejmp.2018.05.006 29891089

[6] Choi MS, Choi BS, Chung SY, Kim N, Chun J, Kim YB, et al. Clinical evaluation of atlas- and deep learning-based automatic segmentation of multiple organs and clinical target volumes for breast cancer. Radiother Oncol. 2020;153:139–45. 10.1016/j.radonc.2020.09.045 32991916

[7] Byun HK, Chang JS, Choi MS, Chun J, Jung J, Jeong C, et al. Evaluation of deep learning-based autosegmentation in breast cancer radiotherapy. Radiat Oncol. 2021;16:1–8. 10.1186/s13014-021-01923-1 34649569 PMC8518257

[8] Liu Z, Liu F, Chen W, Tao Y, Liu X, Zhang F, et al. Automatic segmentation of clinical target volume and organs-at-risk for breast conservative radiotherapy using a convolutional neural network. Cancer Manag Res. 2021;13:8209–17. 10.2147/CMAR.S330249 34754241 PMC8572021

[9] Chung SY, Chang JS, Choi MS, Chang Y, Choi BS, Chun J, et al. Clinical feasibility of deep learning-based auto-segmentation of target volumes and organs-at-risk in breast cancer patients after breast-conserving surgery. Radiat Oncol. 2021;16:1–10. 10.1186/s13014-021-01771-z 33632248 PMC7905884

[10] Almberg SS, Lervåg C, Frengen J, Eidem M, Abramova TM, Nordstrand CS, et al. Training, validation, and clinical implementation of a deep-learning segmentation model for radiotherapy of loco-regional breast cancer. Radiother Oncol. 2022;173:62–8. 10.1016/j.radonc.2022.05.018 35618100

[11] McIntosh C, Conroy L, Tjong MC, Craig T, Bayley A, Catton C, et al. Clinical integration of machine learning for curative-intent radiation treatment of patients with prostate cancer. Nat Med. 2021;27:999–1005. 10.1038/s41591-021-01359-w 34083812

[12] Barragán-Montero A, Bibal A, Dastarac MH, Draguet C, Valdés G, Nguyen D, et al. Towards a safe and efficient clinical implementation of machine learning in radiation oncology by exploring model interpretability, explainability and data-model dependency. Phys Med Biol. 2023;67:11TR01. 10.1088/1361-6560/ac678a PMC9870296 35421855

[13] Bakx N, Rijkaart D, van der Sangen M, Theuws J, van der Toorn PP, Verrijssen AS, et al. Clinical evaluation of a deep learning segmentation model including manual adjustments afterwards for locally advanced breast cancer. Tech Innov Patient Support Radiat Oncol. 2023;26:0–5. 10.1016/j.tipsro.2023.100211 PMC10205480 37229460

[14] Hindocha S, Zucker K, Jena R, Banfill K, Mackay K, Price G, et al. Artificial intelligence for radiotherapy auto-contouring: Current use, perceptions of and barriers to implementation. Clin Oncol. 2023;35:219–26. 10.1016/j.clon.2023.01.014 36725406

[15] Hesso I, Kayyali R, Dolton DR, Joo K, Zacharias L, Charalambous A, et al. Cancer care at the time of the fourth industrial revolution: an insight to healthcare professionals’ perspectives on cancer care and artificial intelligence. Radiat Oncol. 2023;18:1–16. 10.1186/s13014-023-02351-z 37814325 PMC10561443

[16] Çiçek Ö, Abdulkadir A, Lienkamp SS, Brox T, Ronneberger O. 3D U-net: Learning dense volumetric segmentation from sparse annotation. Med Image Comput Comput Assist Interv. 2016;9901:424–32. 10.1007/978-3-319-46723-8_49

[17] Nikolov S, Blackwell S, Zverovitch A, Mendes R, Livne M, de Fauw J, et al. Clinically applicable segmentation of head and neck anatomy for radiotherapy: Deep learning algorithm development and validation study. J Med Internet Res. 2021;23(7):e26151. 10.2196/26151 34255661 PMC8314151

[18] Mikalsen SG, Skjøtskift T, Flote VG, Hämäläinen NP, Heydari M, Rydén-Eilertsen K. Extensive clinical testing of deep learning segmentation models for thorax and breast cancer radiotherapy planning. Acta Oncol (Madr). 2023;62:1184–93. 10.1080/0284186X.2023.2270152 37883678

